# Identification of candidate loci for adaptive phenotypic plasticity in natural populations of spadefoot toads

**DOI:** 10.1002/ece3.6602

**Published:** 2020-07-24

**Authors:** Nicholas A. Levis, Emily M. X. Reed, David W. Pfennig, Martha O. Burford Reiskind

**Affiliations:** ^1^ Department of Biology University of North Carolina Chapel Hill NC USA; ^2^ Department of Biological Sciences North Carolina State University Raleigh NC USA

**Keywords:** assessment, ddRADseq, outlier loci, phenotypic plasticity, population genomics, spadefoot toad

## Abstract

Phenotypic plasticity allows organisms to alter their phenotype in direct response to changes in the environment. Despite growing recognition of plasticity's role in ecology and evolution, few studies have probed plasticity's molecular bases—especially using natural populations. We investigated the genetic basis of phenotypic plasticity in natural populations of spadefoot toads (*Spea multiplicata*). *Spea* tadpoles normally develop into an “omnivore” morph that is favored in long‐lasting, low‐density ponds. However, if tadpoles consume freshwater shrimp or other tadpoles, they can alternatively develop (via plasticity) into a “carnivore” morph that is favored in ephemeral, high‐density ponds. By combining natural variation in pond ecology and morph production with population genetic approaches, we identified candidate loci associated with each morph (carnivores vs. omnivores) and loci associated with adaptive phenotypic plasticity (adaptive vs. maladaptive morph choice). Our candidate morph loci mapped to two genes, whereas our candidate plasticity loci mapped to 14 genes. In both cases, the identified genes tended to have functions related to their putative role in spadefoot tadpole biology. Our results thereby form the basis for future studies into the molecular mechanisms that mediate plasticity in spadefoots. More generally, these results illustrate how diverse loci might mediate adaptive plasticity.

## Graphical abstract

By combining natural variation in pond ecology and ecomorph production with population genetic approaches, we identified candidate loci associated with morph (carnivores vs. omnivores) and loci associated with adaptive phenotypic plasticity (adaptive vs. maladaptive morph choice) in spadefoot toad tadpoles. We identified 16 candidate genes that tended to have functions related to their putative role in spadefoot tadpole biology, and most of these genes were associated with adaptive plasticity rather than morph.

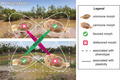

## 1 INTRODUCTION

Phenotypic plasticity—the ability of an individual organism to alter its phenotype in direct response to changes in its environment (hereafter, “plasticity”)—is increasingly thought to play diverse roles in ecology and evolution (Forsman, 2015; Hendry, 2016; Pfennig et al., 2010; West‐Eberhard, 2003; Whitman & Agrawal, 2009). Such roles range from enabling populations to persist in novel or changing environments (Bradshaw, 1965; Fox, Donelson, Schunter, Ravasi, & Gaitán‐Espitia, 2019; Lande, 2009; Snell‐Rood, 2013; Yeh & Price, 2004) to facilitating the evolutionary origins of novel, complex phenotypes (Levis & Pfennig, 2016, 2019b; Moczek et al., 2011; Pfennig et al., 2010; Price, Qvarnstrom, & Irwin, 2003; Scheiner, Barfield, & Holt, 2017; West‐Eberhard, 2003; Wund, 2012). Yet, despite these important roles, little is known of plasticity's underlying proximate mechanisms. This is especially true for *adaptive* plasticity, which we define as a change in a phenotype that occurs in response to a specific environmental signal or cue and that enhances fitness (sensu Stearns, 2014). Clarifying the molecular mechanisms underlying adaptive plasticity is crucial for ultimately understanding its downstream impacts on ecology and evolution (Gilbert & Epel, 2015; Levis & Pfennig, 2020). [NB: We treat plasticity as synonymous/encompassing G × E, because plasticity naturally requires an interaction between an organism's genes and its environment (i.e., purely environmental effects are not biologically plausible), and because natural populations often show variation in plasticity among genotypes or individuals (Falconer & Mackay, 1996; Sultan & Stearns, 2005).]

As a first step toward illuminating the proximate mechanisms of adaptive plasticity, consider that adaptive plasticity typically entails two main stages (West‐Eberhard, 1992, 2003; Whitman & Agrawal, 2009; Windig, de Kovel, & de Jong, 2004). First, the organism must *assess* which of a series of plausible alternative phenotypes that it could produce will likely be associated with the highest fitness, given both the external environmental conditions and the individual's current state (e.g., its size, age, sex). Such assessment involves systems for receiving information from the environment and systems for relaying that information to parts of the organism where it can be processed (West‐Eberhard, 2003). Second, for adaptive plasticity to occur, assessment must be followed by a developmental *response* that results in production of a (putatively adaptive) phenotype. This stage often involves a complex set of mechanisms that result in production of a behavioral, physiological, or morphological phenotype that is suited to current environmental conditions (Beldade, Mateus, & Keller, 2011; Lafuente & Beldade, 2019).

The prevailing view is that plasticity (assessment and response) and the phenotype produced by plasticity are likely regulated by different loci (Lafuente, Duneau, & Beldade, 2018; Ørsted, Rohde, Hoffmann, Sørensen, & Kristensen, 2018; Scheiner & Lyman, 1991; Schlichting & Pigliucci, 1993; Windig et al., 2004). However, because research into the genetic bases of plasticity is in its infancy (reviewed in Lafuente & Beldade, 2019), there are few tests of this prediction. Moreover, while laboratory studies have greatly advanced our understanding of plasticity's molecular basis (e.g., Casasa & Moczek, 2018; Gibson & Hogness, 1996; Lafuente et al., 2018; Parsons et al., 2016; Ragsdale, Müller, Rödelsperger, & Sommer, 2013; Suzuki & Nijhout, 2008), few (if any) studies have sought to unravel the genetic basis of plasticity using natural populations. Here, we identify candidate loci regulating adaptive phenotypic plasticity in natural populations of spadefoot toads.

**Figure 1:**
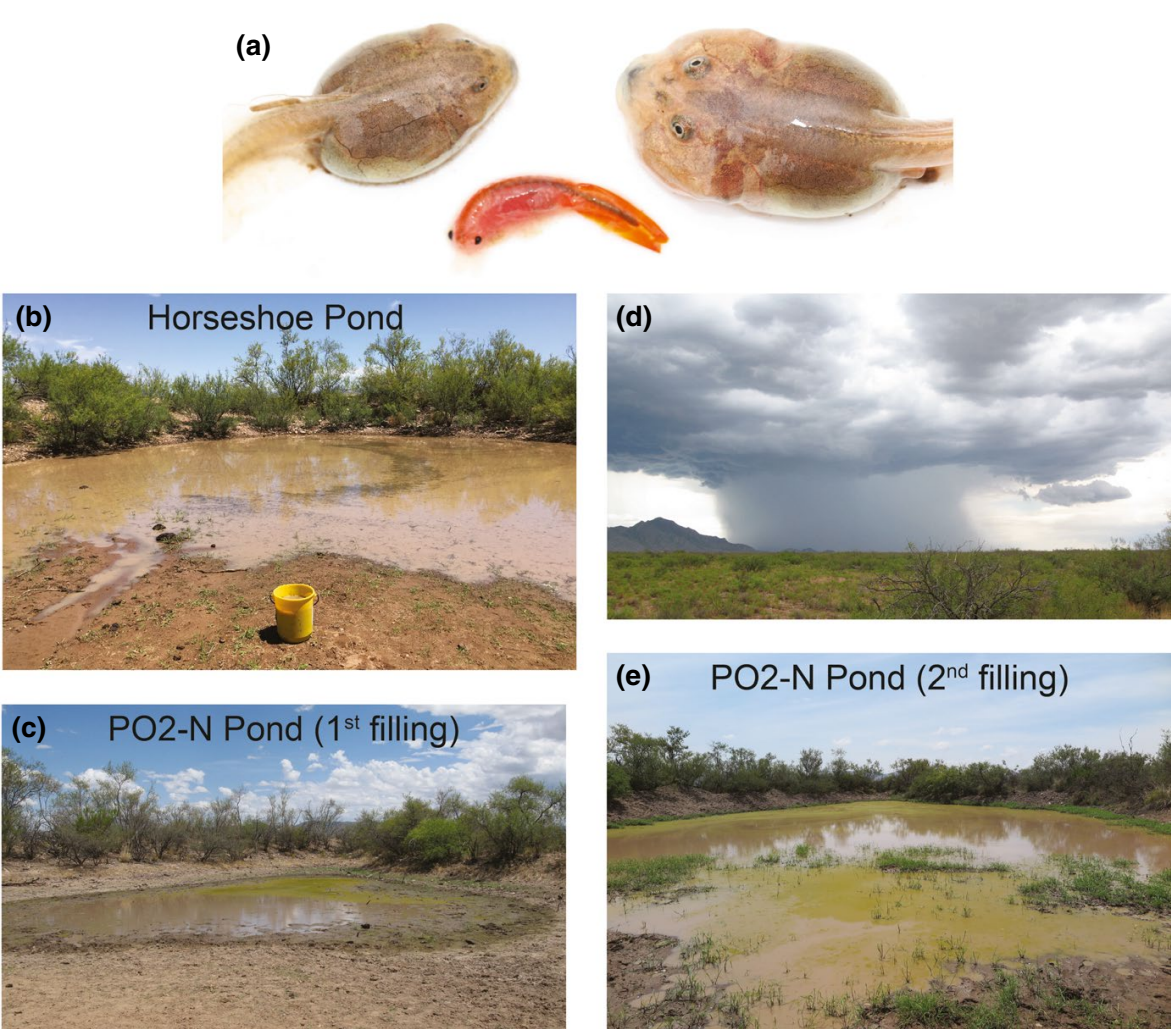
Study system. (a) Mexican spadefoot toads, *Spea multiplicata*, produce alternative, environmentally induced morphs: a slower developing omnivore morph (left) and a more rapidly developing carnivore morph (right), which is induced by, and specializes on, animal prey, such as fairy shrimp (center). The adults of this species emerge for only a few weeks each year to feed and to breed in temporary, rain‐filled ponds, such as (b) Horseshoe Pond and (c) PO2‐N Pond. (d) Highly localized summer thunderstorms can sometimes (e) refill these ponds, as occurred during our study in PO2‐N Pond (but not the nearby and initially similar Horseshoe Pond)

We focused on Mexican spadefoot toad (*Spea multiplicata*) tadpoles, which have evolved a conspicuous form of phenotypic plasticity. Normally, these tadpoles feed on detritus and microorganisms and, consequently, develop small jaw muscles, smooth keratinized mouthparts, numerous denticle rows, and a long gut, which enable them to feed on these resources (Pfennig, 1990; Pomeroy, 1981). However, in contrast with this default “omnivore” morph, if a young tadpole consumes fairy shrimp or other tadpoles (Levis, de la Serna Buzon, & Pfennig, 2015; Pfennig, 1990, 1992b), it can use its inherent plasticity to develop into an alternative, environmentally induced “carnivore” morph. This morph develops features that enable it to specialize on large, mobile prey (Paull, Martin, & Pfennig, 2012). Specifically, the carnivore morph is characterized by large jaw muscles, notched keratinized mouthparts, few denticle rows, and a short gut (Figure 1a). The frequency with which carnivores are produced––and how extreme these carnivores are––varies among species, populations, and even among different sibships in the same population (Martin & Pfennig, 2011; Pfennig, 1999; Pfennig & Murphy, 2000), suggesting underlying heritable (possibly genetic) variation in propensity to produce and express the carnivore phenotype.

Generally, carnivores are favored in shrimp‐rich, short‐duration ponds (because carnivores develop faster), whereas omnivores are favored in shrimp‐poor, longer‐duration pond (because, given sufficient time to develop, omnivores can consume a wider range of resources; Paull et al., 2012; Pfennig, 1990, 1992a). However, because of the highly unpredictable and localized nature of rainfall patterns in the regions where spadefoots breed, there is considerable year‐to‐year variation in the duration of any given pond and therefore the abundance of shrimp therein (Pfennig, 1990, 2007). Thus, in this system, selection has presumably favored the ability to use plasticity to assess and respond to a highly variable environment. Indeed, if pond conditions change (e.g., because of a second pond filling), individuals can even reassess the environment and potentially switch to the alternative morph (Pfennig, 1992a). Yet, not all omnivores are alike, nor are all carnivores, meaning that there is often morphological variation present in a population in the degree to which each of these two extreme phenotypes is expressed (Levis, Martin, O’Donnell, & Pfennig, 2017; Martin & Pfennig, 2010). Thus, selection has also presumably favored the ability to produce the appropriate “type” of omnivore or carnivore, given pond conditions.

By taking advantage of such variation between naturally occurring populations in which morph was favored, we were able to employ population genetic analyses to identify loci associated with each of these two morphs as well as those associated with the ability to express adaptive plasticity in the first place. As predicted by theory (see above), we found candidate loci associated with these alternative morphs and candidate genes associated with the ability to express adaptive plasticity. Our results thereby provide a basis for understanding the molecular mechanisms that mediate plasticity in spadefoots specifically and for illustrating how diverse loci might be deployed to mediate plasticity more generally.

## 2 METHODS

### 2.1 Goals

Our study had two goals. First, we sought to identify candidate loci that were differentially associated with alternative phenotypes that are produced by plasticity (i.e., loci associated with carnivores vs. omnivores). Second, we also sought to identify candidate loci that were differentially associated with individuals’ ability to assess and respond adaptively to their environment through adaptive plasticity (i.e., loci associated with adaptive versus maladaptive morph choice). Regarding the first goal, we expected to find candidate loci differentially associated with carnivores versus omnivores––despite the important role that the environment plays in influencing which morph an individual becomes––because of *a priori* evidence of underlying genetic variation in propensity to produce and express the carnivore phenotype (see pentultimate paragraph in the Introduction). Regarding the second goal, we expected to find candidate loci differentially associated with adaptive versus maladaptive morph choice because of *a priori* evidence that different ecological conditions favor carnivores versus omnivores (as also described in the Introduction and below).

To accomplish both goals, we collected DNA from equal numbers of carnivore and omnivore tadpoles from two nearby naturally occurring ponds that differed in which morph was ultimately favored by selection, and combined this “natural experiment” with double‐digest restriction associated DNA sequencing (ddRADseq) and various population genetic tools and analyses (summarized in Table 1). At loci associated with phenotype, we expected carnivores in both ponds to be more similar to each other than to omnivores and omnivores to be more similar to each other than to carnivores. Conversely, at loci associated with plasticity, we expected carnivores in the carnivore‐favored pond to be similar to omnivores in the omnivore‐favored pond.

**Table 1:** Summary of methods used in this study and the number of loci (or genes) identified at each step

Purpose	Method	Number of loci or genes
Loci generation	STACKS	20,328
Outlier detection	LOSITAN and DAPC	113
Allele frequency variation	Fisher's exact test with fdr correction	12 (0); 60 (49)
Genome mapping	Bowtie2	2;14

Note

We first generated loci using STACKS and detected outliers using LOSITAN and DAPC. For these outliers, we then evaluated differences in allele frequencies based on phenotype (or morph; left of semicolon) and plasticity (right of semicolon). Values in parentheses denote the number of significant loci following multiple testing correction. Finally, we mapped reads containing candidate loci (12 loci for phenotype; 49 loci for plasticity) to the *S. multiplicata* reference genome and identified nearby genes.

### 2.2 Collection sites

We focused on two populations of *S. multiplicata* that live in and around two temporary ponds in the San Simon Valley near Portal, Arizona, USA: “Horseshoe” Pond (31.9389, −109.0864; Figure 1b) and “PO2‐N” Pond (31.9142, −109.0836; Figure 1c). These two ponds are 2.8 km apart and similar ecologically. Moreover, *S. multiplicata* have been observed to breed in these ponds for at least the past 45 years (Pomeroy, 1981). The two ponds are “stock tanks”: reservoirs formed when humans constructed earthen dams across gullies to catch runoff and hold water temporarily for cattle. These two tanks, like most others in the San Simon Valley, were likely built in the early 1930s (Gillespie, 2014). Thus, the two populations could have been first colonized 45 spadefoot generations ago, possibly from the same source population (a likely candidate is the nearby––but now permanently dry––San Simon Ciénega). Given the lack of physical barriers, individuals might occasionally migrate between Horseshoe Pond and PO2‐N Pond, especially in wet years, when traversing the normally uninhabitable desert scrub surrounding these ponds is more tenable. However, previous work revealed significant population genetic structure among populations of *S*. *multiplicata* in the San Simon Valley (Pfennig & Rice, 2014; Rice & Pfennig, 2010), which makes sense given that opportunities for dispersal are rare: adult *Spea* spend much of the year underground, emerging only for a few weeks during the summer rainy season to breed and feed before burrowing underground again (Bragg, 1965).

Both Horseshoe Pond and PO2‐N Pond filled from the same thunderstorm on the afternoon 2 July 2016 and were initially similar in size (~50 m^2^ in area and 15 cm in maximum depth). That evening, we observed about 12 female *S*. *multiplicata* and several Couch's spadefoot toads, *Scaphiopus couchii*, breeding in each pond. Because all breeding took place on the same night (July 2; no more breedings were observed in either pond), and because *Spea* eggs hatch rapidly (in about 2 days), all tadpoles in both ponds were of the same age and initially experienced similar environments. Moreover, by July 14 (10 days after the tadpoles hatched), we observed that a similar fraction of *S*. *multiplicata* tadpoles in each pond (~50%) had developed into carnivores (Levis et al., 2017).

On July 16 (12 days after the tadpoles hatched), a second thunderstorm flooded PO2‐N Pond, doubling its original size (i.e., to ~100 m^2^ in area). Such a situation is not unusual: during the summer, the San Simon Valley experiences thunderstorms, which produce heavy, but localized rain (Figure 1d). Consequently, while any given pond may receive runoff from such storms, another nearby pond might not. This particular storm refilled PO2‐N Pond––but not Horseshoe Pond––thereby transforming PO2‐N Pond from a small, ephemeral pond (Figure 1c) into a larger, longer‐duration pond (Figure 1e). The increase in water volume also greatly reduced fairy shrimp and *Sc. couchii* tadpole densities (the primary resources of carnivores). Indeed, shortly thereafter, we began to observe emaciated‐looking carnivores in PO2‐N Pond (but not in Horseshoe).

This divergence between these two ponds in their ecology is important. The second filling allowed us to decouple a tadpole's phenotype (morph) from its ability to assess and respond adaptively to changing conditions (plasticity). Given the initial equal frequencies of carnivores and omnivores in both ponds, with the second filling of PO2‐N, the two ponds diverged in selective environment, with PO2‐N transforming from a short‐duration, high‐shrimp density pond, which (like in Horseshoe) favored carnivores, into a long‐duration, low‐shrimp density pond, which favored omnivores. Typically, long‐duration, shrimp‐poor ponds produce few carnivores (Pfennig, 1990). However, because PO2‐N initially had high shrimp densities, it started off by producing abundant carnivores. Consequently, the second filling allowed some––but not all––carnivores in PO2‐N to adjust their phenotype adaptively via plasticity; specifically, *to switch from a carnivore to an omnivore*. [NB: Controlled experiments have shown that carnivores can indeed revert back into omnivores if environmental conditions change, such as a dramatic reduction in the availability of shrimp or tadpole prey (Pfennig, 1992a, 1992b; Pomeroy, 1981). Moreover, there was ample time between when the second filling occurred (July 16) and when the tadpoles were collected (July 30; see next section) for these individuals to switch morphology. However, it is possible that some PO2‐N carnivores may have died before they could revert to omnivores.]

In sum, the fact that both morphs were equally abundant in both ponds––even though the ponds ended up being very different in which morph was favored by selection (omnivores in PO2‐N and carnivores in Horseshoe; confirmed by de la Serna Buzón, 2019 as described in the next section)––*provided us with an ideal opportunity to separate phenotype from plasticity*.

### 2.3 Sample collection

**Figure 2:**
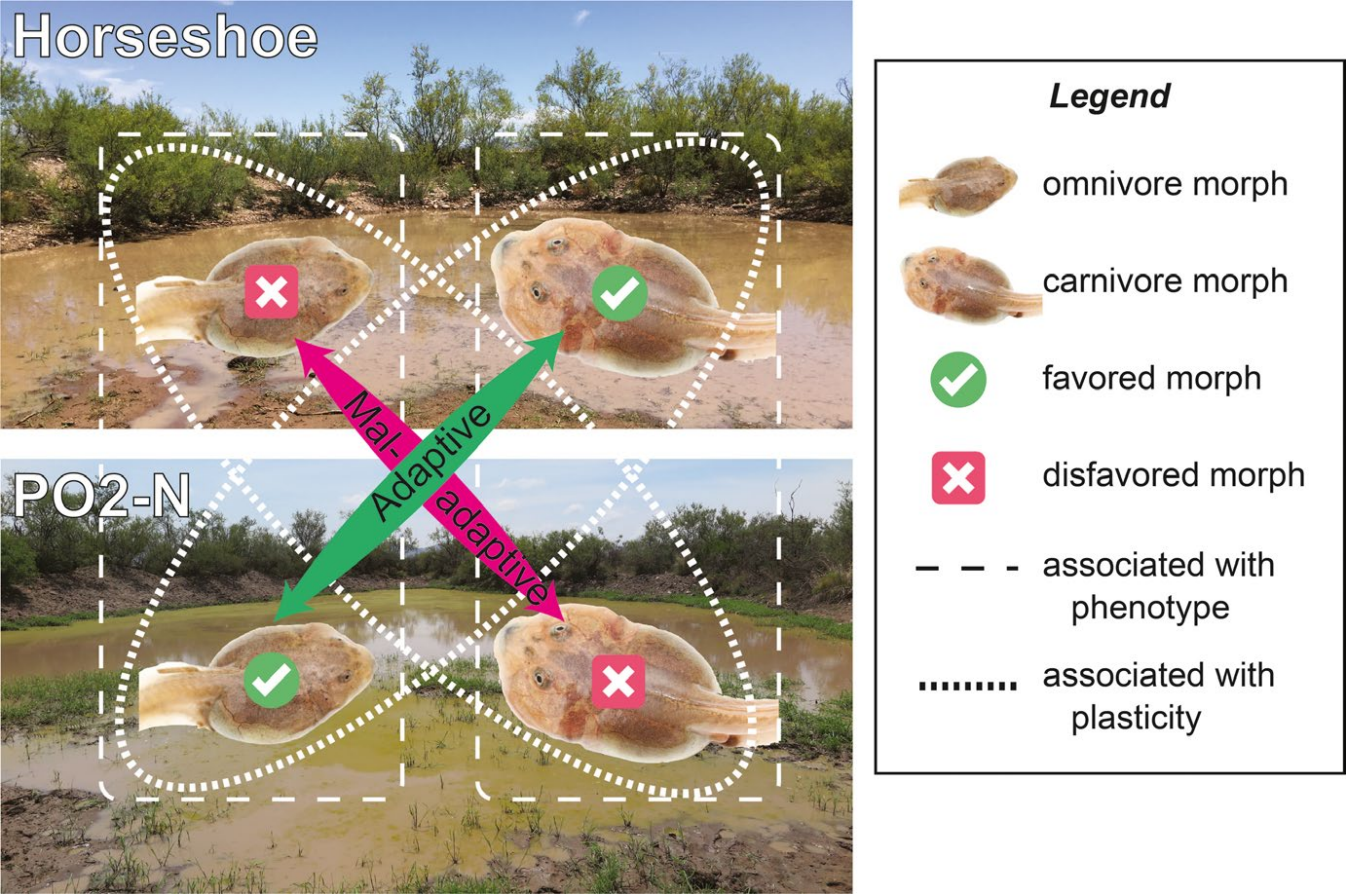
Study design. Although Horseshoe Pond and PO2‐N Pond were initially similar, a second refilling of PO2‐N Pond caused the two ponds to diverge in hydroperiod and resource density, thereby resulting in different tadpole morphs being favored in each pond. We sought to identify candidate loci differentially associated with phenotype (i.e., carnivores vs. omnivores) and those differentially associated with plasticity (i.e., adaptive vs. maladaptive morph choice). See text for additional explanation

We sought to identify candidate loci differentially associated with carnivores versus omnivores and candidate loci differentially associated with adaptive versus maladaptive morph choice (i.e., become a carnivore in Horseshoe Pond and an omnivore in PO2‐N Pond versus an omnivore in Horseshoe Pond and a carnivore in PO2‐N Pond, respectively; Figure 2). We therefore needed to sample late‐stage tadpoles of each morph from each pond.

On July 26 (22days after the tadpoles hatched), we detected in Horseshoe Pond a few individuals approaching metamorphosis (as evidenced by a forelimb protruding from their body). We therefore immediately sampled about 100 late‐stage carnivores and 100 late‐stage omnivores from Horseshoe. In doing so, we categorized each tadpole sampled as either a carnivore or an omnivore by visually characterizing the shape of its head and mouthparts (see Levis et al., 2015 and references therein); all morph assignments were unambiguous (we collected late‐stage tadpoles [Gosner (1960) stages 40–41], rather than metamorphs [Gosner (1960) stage 46], because the two morphs are nearly indistinguishable morphologically at metamorphosis.) These tadpoles were transported to the nearby Southwestern Research Station, where 50 tadpoles of each morph were placed into separate wading pools (1.8 m diameter filled 15 cm deep with well water) and provided with live fairy shrimp and detritus from their natal pond. After 3 days, they were placed (in groups of 10 same‐morph individuals) into 1.6‐L plastic bags filled with pond water and shipped overnight to our laboratory at the University of North Carolina.

PO2‐N tadpoles developed more slowly (presumably because food was less dense following the second filling), so we therefore collected carnivores and omnivores from PO2‐N Pond on July 30 (26 days after hatching). Otherwise, PO2‐N carnivores and omnivores were treated the same as those from Horseshoe Pond. Although some omnivores sampled from PO2‐N Pond were undoubtedly omnivores from birth (having never actually made the *initial* adaptive choice to become carnivores before reverting back to omnivores following the second filling), our anecdotal data suggest that many of these omnivores had indeed switched from carnivore to omnivore: on many of the omnivores that we sampled, we noted omnivore‐like jaw musculature and *carnivore*‐like gut length, which is diagnostic of an individual that had switched from carnivore to omnivore (the gut does not revert as readily as jaw muscle; Pfennig, 1992b). Even if we acknowledge that some of the omnivores from PO2‐N Pond were likely omnivores all along, these individuals had (after the second filling) made the adaptive “decision” to remain as omnivores. More importantly, given the dramatic change in resource hydroperiod and resource abundance in PO2‐N, the carnivores that we sampled from this pond had clearly made a *maladaptive* “decision” to remain as carnivores. Thus, while our PO2‐N omnivore sample was “noisy” (because it likely contained a mix of individuals that had made adaptive and maladaptive morph choices), the remaining *three* samples (PO2‐N carnivores and Horseshoe carnivores and omnivores) would have been considerably less noisy regarding which individuals made adaptive versus maladaptive morph choices (see also Figure 2).

Back in the laboratory, tadpoles and metamorphs were kept separate by population and morph, but were treated the same otherwise (de la Serna Buzón, 2019). As soon as an individual reached metamorphosis, we took a toe clip from the individual, placed the toe clip in a labeled vile filled with ethanol, and stored the vial in a freezer kept at −80°C.

We then reared all surviving individuals to sexual maturity for a separate study (de la Serna Buzón, 2019). As part of this second study, we measured each individual's: (a) size at metamorphosis; (b) age at metamorphosis; (c) size at sexual maturity; (d) age at sexual maturity; and (e) survival to 400 days old (the age at which most individuals had achieved sexual maturity). Size at maturity was relevant, because, in *Spea*, larger males have higher mating success (Pfennig, 2008), and larger females have higher fecundity (Pfennig & Pfennig, 2005). Measuring survival, age, and size at sexual maturity enabled us to confirm our *a priori* expectation that selection favored carnivores in Horseshoe Pond and omnivores in PO2‐N Pond (de la Serna Buzón, 2019). For the final sample sizes, we had four sample types: Horseshoe carnivores (C_Horse_; *n* = 47) and omnivores (O_Horse_; *n* = 44) and PO2‐N carnivores (C_PO2‐N_; *n* = 21) and omnivores (O_PO2‐N_; *n* = 20).

Finally, the fact that we sampled individuals from only two ponds might be criticized on the grounds that we had no replication of each pond type. However, we were not interested in exploring a possible association between a *particular* pond type (i.e., environment) and a specific set of candidate genes. We were merely interested in establishing whether or not different candidate genes were associated with: (a) carnivores versus omnivores, and (b) those individuals that made the overall adaptive choice in which morph to become (i.e., omnivores in PO2‐N and carnivores in Horseshoe) versus those that made the overall maladaptive choice (i.e., carnivores in PO2‐N and omnivores in Horseshoe). Thus, we treated individuals of each morphotype within each pond as independent replicates.

### 2.4 DNA extraction and double‐digest RAD sequencing (ddRADseq) library building

We extracted genomic DNA from juvenile toad toe clips. We used a Qiagen DNeasy Blood & Tissue Kits (Qiagen Inc.) and quantified template DNA using a fluorometer (Qubit 2.0; Invitrogen) following both manufacturer's protocols, with the exception that we used 30 μl of proteinase K, digested the samples for 72 hr, and eluted in H_2_O to allow for subsequent concentration of DNA if needed.

Double‐digest RAD sequencing libraries were built using the enzyme pair *Sph*I and *Mlu*CI according to the protocol and methods described in Burford Reiskind et al. (2016) and Burford Reiskind, Labadie, Bargielowski, Lounibos, and Reiskind (2018). To facilitate multiplexing and assignment of individual barcodes (Burford Reiskind et al., 2016), we distributed our samples across four libraries and used 200 ng of template DNA per individual. We conducted single‐end sequencing of 100 bp fragments on two lanes of the HiSeq 4000 at the University of Oregon GC3F facility. To enable unique identification of samples (Burford Reiskind et al., 2016), we ran two libraries per sequencing lane and had samples evenly distributed across lanes.

### 2.5 Initial quality control and SNP detection

We performed demultiplexing, read processing, and SNP detection using STACKS v1.24 (Catchen, Hohenlohe, Bassham, Amores, & Cresko, 2013) in accordance with Burford Reiskind et al. (2016) and Burford Reiskind et al. (2018). SNP detection was done using the *denovo* pipeline (*denovo.pl*) with the following parameters: *m* = 5 (minimum stack depth), *M* = 3 (mismatches allowed between reads within an individual for creating loci), and *n* = 2 (mismatches allowed between loci when combining them in a catalog for all individuals) (Catchen et al., 2013). Following SNP detection, we ran the STACKS population pipeline (populations) independently for Horseshoe and PO2‐N Ponds (treating carnivores and omnivores as separate populations within each geographic population) with the following parameters: minimum number of stacks per individual at a locus (*m* = 5), number of populations loci present in (*p* = 2), proportion of individuals within a population that have these loci (*r* = 0.90), and appropriate output files for downstream analyses. We ran the output of the STACKS pipeline in the PLINK v1.19 format (Purcell et al., 2007) and used the program PGDSPIDER v2.1.1.0 (Lischer & Excoffier, 2012) to transform the PLINK dataset into various formats required by the following software: GenePop v4.2 (Rousset, 2008), LOSITAN (Antao, Lopes, Lopes, Beja‐Pereira, & Luikart, 2008), and discriminant analysis of principal components (DAPC) implemented in R (Jombart, 2008). Prior to downstream analyses, we used PLINK to filter out monomorphic loci (‐maf 0.01) and remove loci with too much missing data (‐geno 0.25). To assess linkage disequilibrium (LD) among loci, we used the R package SNPRelate (Zheng et al., 2012). Because linkage was generally low (the median absolute LD among our loci was 0.085), and because linkage did not decrease much after setting fairly stringent cutoffs (e.g., forcing an LD threshold among loci to be ≤0.1 resulted in a median LD of 0.057), we retained all SNPs for downstream analyses. We made a final assessment of linkage when we aligned reads with outlier loci to the draft genome (see section 2.6 below). Finally, we assessed genetic differentiation (*F*_ST_) between carnivores and omnivores in each geographic population separately and among all four sample types simultaneously using GenePop (MCMC parameters: 20,000 dememorization, 500 batches, 10,000 iterations per batch).

### 2.6 Outlier loci and candidate genes

Recall that the main goal of this study was to identify candidate loci that differentiate carnivores and omnivores (phenotype) and that differentiate individuals’ ability to detect and respond to environmental change (plasticity). That is, we were interested in finding candidate loci that discriminate between carnivores (C_Horse_ + C_PO2‐N_) and omnivores (O_Horse_ + O_PO2‐N_) and that discriminate between individuals that adaptively assess and respond to their environment (C_Horse_ + O_PO2‐N_) and those that do not (O_Horse_ + C_PO2‐N_). We utilized two approaches to detect outlier loci between morphs and among morph‐population groups knowing that alternative morphs were favored in the different geographic populations. Both methods of outlier detection work best when there is low genetic differentiation between populations based on drift, a condition we confirmed prior to outlier detection (see section 3.1).

Our first approach detected outlier loci between morphs in each population independently using 10 reps of 1,000,000 simulations in LOSITAN. We applied false discovery rate (fdr) correction factor of the p‐value of 0.1 using LOSITAN’s main algorithm FDIST2 (Beaumont & Nichols, 1996; Burford Reiskind et al., 2018). Given that we were comparing only a few groupings, we chose LOSITAN to detect outlier loci because it is robust to issues associated with small numbers of populations (see Tigano, Shultz, Edwards, Robertson, & Friesen, 2017).

As a second outlier detection method, we conducted a DAPC on all four morph‐population groups in the R package ADEGENET because it is able to discriminate between complex population models (e.g., those with hierarchical structure; Jombart, Devillard, & Balloux, 2010; Tigano et al., 2017). To obtain the optimum number of principal components to retain in the DAPC, we performed a cross‐validation method using a 95% training set and 500 replicates, and conducted it with the chosen number of principal components using average linkage clustering method to set a threshold for outlier loci. We only included those loci identified as outliers in each population using LOSITAN or that were detected using DAPC on all four morph‐population groups. Thus, we compiled outlier loci detected using both methods into a single database for downstream investigation. The number of loci we detected using this approach (see section 3.2 below and Table 1) is consistent with other studies (e.g., Burford Reiskind et al., 2018; Tigano et al., 2017).

For each outlier, we looked at the pattern of allele frequencies to determine whether allele frequencies varied by phenotype and/or plasticity. Specifically, for each locus, we determined the frequency of both alleles in each population‐morph group and then tested for significant differences (using Fisher's exact test followed by fdr correction for multiple testing) based on: (a) morph (C_Horse_ + C_PO2‐N_ vs. O_Horse_ + O_PO2‐N_) and/or (b) plasticity (C_Horse_ + O_PO2‐N_ vs. O_Horse_ + C_PO2‐N_). Essentially, we asked whether any alleles were associated with (a) morph, regardless of which pond the individual came from (suggesting that these alleles were associated with “phenotype”), and (b) the selectively favored morph, regardless of which pond the individual came from (suggesting that these alleles were associated with “plasticity”).

Loci that were identified as significantly different in each contrast (Dryad dataset: https://doi.org/10.5061/dryad.h9w0vt4fj) were then mapped to the *S. multiplicata* reference genome (Seidl et al., 2019) using the “very sensitive local” setting in Bowtie2 (Langmead & Salzberg, 2012). Following mapping, we identified candidate genes for phenotype and/or plasticity as those for which an outlier locus occurred within the gene itself or where an outlier mapped within 10 kb upstream or downstream of the gene.

## 3 RESULTS

### 3.1 SNP detection

For Horseshoe and PO2‐N Ponds, the STACKS pipeline generated 39,294 and 34,478 loci, respectively. Of these, 20,328 were shared between the two populations. In each population, *F*_ST_ between morphs was 0 and not significant. For all four morph‐population groups together, STACKS generated 22,237 loci and all pairwise *F*_ST_ values were 0 (except between C_PO2‐N_ and O_Horse_; *F*_ST_ = 0.0009) and not significantly different. Thus, there was little genetic differentiation among our population‐morph groups.

### 3.2 Outlier loci and candidate genes

LOSITAN identified 1,190 outlier loci between morphs in Horseshoe and 2,935 outlier loci between morphs in PO2‐N. The populations overlapped at 76 of these outliers. DAPC detected 37 additional outliers among morph‐population groups. In total, we had a list of 113 candidate outliers for allele frequency assessment.

Of the 113 outlier loci we identified, only 12 loci were significantly different in allele frequencies between morphs, but this reduced down to 0 after correcting for multiple testing (Table S1). More loci were associated with plasticity: 60 loci were significantly different between groups that adaptively assessed and responded to their environment versus those that did not (i.e., these loci differed in the expression of adaptive plasticity). After multiple testing correction, this reduced down to 49 loci (Table S1).

To identify any candidate morph (phenotype) genes, we mapped the 12 loci whose allele frequencies significantly differed by morph (even though these differences were no longer significant after multiple testing correction). These loci were located on 10 unique reads and mapped to two genes on separate scaffolds whose functions tended to be structural (Table 2). Specifically, these genes were associated with stabilization and support of muscle fibers and acting as a molecular chaperone responsible for the organization of intermediate filaments.

**Table 2:** List of candidate genes identified in this study, and how their functions may be relevant to their classification as being associated with phenotype versus plasticity

Gene symbol	Gene name	Classification	Relevant function(s)
DMD	Dystrophin	Phenotype	Helps stabilize and support muscle fibers
SACS	Sacsin molecular chaperone	Phenotype	Contributes to organization of intermediate filaments
AFG3L2	AFG3 like matrix AAA peptidase subunit 2	Plasticity	Affects mitochondrial protein stability and links mitochondrial metabolism and axonal development
CLIP1	CAP‐Gly domain‐containing linker protein 1	Plasticity	Affects neuronal development
CUL9	Cullin 9	Plasticity	Affects cell proliferation, senescence, and apoptosis
FAP	Fibroblast activation protein alpha	Plasticity	Regulates cell proliferation and differentiation
GPR83	G‐protein‐coupled receptor 83	Plasticity	Affects learning, behavior, and metabolic regulation
IBTK	Inhibitor of Bruton tyrosine kinase	Plasticity	Regulates signal transduction
JUN	Jun proto‐oncogene, AP‐1 transcription factor subunit	Plasticity	Helps prevent UV‐induced apoptosis and promotes cellular differentiation and nerve cell regeneration
MAP3K4	Mitogen‐activated protein kinase kinase kinase 4	Plasticity	Affects signal transduction of environmental stress
MAST2	Microtubule‐associated serine/threonine kinase 2	Plasticity	Regulates cell (neuron) survival
MED13	Mediator complex subunit 13	Plasticity	Helps regulate transcription
NDUFAF7	NADH:ubiquinone oxidoreductase complex assembly factor 7	Plasticity	Helps maintain electron transport chain
NHLRC1	NHL repeat containing E3 ubiquitin protein ligase 1	Plasticity	Helps regulate glycogen synthesis
SSH1	Slingshot protein phosphatase 1	Plasticity	Regulates smooth muscle cell migration
TENT5A	Terminal nucleotidyltransferase 5A	Plasticity	Helps regulate transcription

For the plasticity loci, we mapped only those 49 loci that passed multiple testing correction. These candidate loci were located on 39 unique reads and mapped to 14 genes (Table 2) across six scaffolds. Reads that mapped to the same scaffold were, on average, 11 Mb apart. The loci assigned to the genes TENT5A and IBTK were within 160 kb of each other, but all other loci were at least 4 Mb away from each other. Seven loci were located within the genes, four loci were within 10 kb (~0.01 cM) upstream, and three loci were within 10 kb downstream. The candidate plasticity genes tended to have functions one would expect for assessment and response. Specifically, these genes were related to learning and behavior, neuron development and function, signal transduction and transcription regulation, cell proliferation, differentiation and migration, and energy management.

## 4 DISCUSSION

The ability to respond adaptively to environmental stimuli––that is, to express adaptive plasticity––is a defining feature of life (Nijhout, 2013). Yet, despite adaptive plasticity's importance, its molecular basis remains poorly understood. Some headway has been made using laboratory‐reared organisms exposed to artificial (but biologically relevant) conditions (reviewed in Lafuente & Beldade, 2019; e.g., Ragsdale et al., 2013). By contrast, clarifying the mechanisms governing how environmental stimuli are translated into developmental outcomes in *natural* populations remains an important frontier (Gilbert & Epel, 2015; Levis & Pfennig, 2020). A first step is to identify loci or genes that are associated with the ability to assess and respond to environmental conditions and/or that are associated with the particular phenotype that develops as result of those conditions. Here, we took such a step by leveraging natural ecological and phenotypic variation among wild populations of spadefoot toad tadpoles. In particular, focusing on the well‐characterized plasticity in these tadpoles (Figure 1a), we sought to identify candidate loci differentially associated with carnivores versus omnivores and candidate loci differentially associated with adaptive versus maladaptive morph choice (i.e., become a carnivore in Horseshoe Pond and an omnivore in PO2‐N Pond versus an omnivore in Horseshoe Pond and a carnivore in PO2‐N Pond, respectively; Figure 2).

The number and type of candidate genes identified in our study may at first glance seem surprising. However, the fact that we found more genes associated with plasticity than with the alternative phenotypes produced by this plasticity is consistent with what is known about this system. In particular, the substantial between‐year and between‐pond variation in pond longevity, resource base, and overall competitive environment (Levis et al., 2017; Levis & Pfennig, 2019a; Paull et al., 2012; Pfennig, 1992a; Pfennig & Simovich, 2002) means that it is unlikely for a particular morph to experience persistent directional selection (but it does happen occasionally; see Levis & Pfennig, 2019a; Pfennig, Rice, & Martin, 2007). However, this same variation in ecology means that the ability to assess and respond appropriately to local conditions *should* be under persistent directional selection. Therefore, it is not surprising that most of the candidate genes that we identified were associated with plasticity instead of phenotype.

Why the particular candidate *phenotype* genes emerged from our analyses is not clear. The most obvious difference between carnivores and omnivores is the presence of enlarged jaw muscles in carnivores (Figure 1a). This difference may partially explain why the gene DMD (which encodes the protein dystrophin) was identified: dystrophin plays a role in stabilization and support of muscle fibers (Blake, Weir, Newey, & Davies, 2002). However, dystrophin could also function in tail muscle: carnivores spend more time swimming than omnivores, and the tail is their primary means of propulsion. The gene SACS (which encodes sacsin protein) may have emerged as a candidate phenotype gene because of its role in cellular protein quality control and possible collaboration with the Hsp70 family of proteins (Anderson, Siller, & Barral, 2011). In particular, this protein may play an important role in maintaining the carnivore (or omnivore) phenotype once it develops rather than contributing to the initial development of the phenotype. More work is needed to disentangle the molecular and physiological differences required to maintain a tadpole's status as a carnivore or omnivore.

The typical functions of our candidate plasticity genes tend to match their putative role in spadefoot tadpole plasticity (i.e., the ability to assess and respond appropriately to current environmental conditions). The genes we identified appear to be associated with four general functions that make sense in the context of switching phenotypes in response to the environment. First, three genes (GPR83, CLIP1, andAFG3L2) are associated with learning, behavior, and/or neuron development (Almajan et al., 2012; Gomes et al., 2016; Larti et al., 2015; Maltecca et al., 2008). Such cognition‐related functions could be related to assessing the dynamic state of the environment and making foraging and developmental decisions depending on the environment. Similarly, as the environment is sampled and assessed, any information gained from that assessment needs to be transduced through developmental pathways and modify development. As it turns out, four of our identified genes served these functions (JUN, TENT5A, MED13, and MAP3K4; Devary, Gottlieb, Lau, & Karin, 1991; Grueter et al., 2012; Kuchta et al., 2016; Takekawa, Posas, & Saito, 1997). Notably, MED13 has been implicated in helping modulate thyroid hormone‐dependent transcription (Grueter et al., 2012), and thyroid hormone, in turn, has been implicated in mediating carnivore development specifically (Pfennig, 1992b) and tadpole development generally (Denver, 1998; Kulkarni & Buchholz, 2012; Kulkarni, Denver, Gomez‐Mestre, & Buchholz, 2017). Moreover, MED13 also plays a role in limiting lipid accumulation (Pospisilik et al., 2010), a key metabolic difference between spadefoot tadpole morphs (de la Serna Buzón, 2019).

If a transduced environmental signal leads to developmental reorganization of the magnitude seen between carnivores and omnivores, then such reorganization likely involves changes in cell proliferation, differentiation, and migration. Our finding five genes with these general functions corroborates this notion (MAST2, SSH1, CUL9, IBTK, and FAP; Fiume et al., 2016; Jia et al., 2018; Lopez & Tait, 2014; Mizuno, 2013; Terrien et al., 2012). Following (or concurrent with) tissue reorganization, metabolic and energy use changes may be expected as part of plastic response in this system given the high dependence on diet as an environmental cue. Therefore, finding a gene (NHLRC1) associated with glycogen synthesis (Worby, Gentry, & Dixon, 2008)—which has previously been discussed in the context of morph differences (de la Serna Buzón, 2019)—and a gene (NDUFAF7) related to maintenance of components needed for aerobic respiration (Rhein, Carroll, Ding, Fearnley, & Walker, 2013) align with known metabolic transitions and differences between morphs (recall that carnivores are more active than omnivores).

At the same time, our study comes with the following three caveats. First, although the general functions of our candidate plasticity genes correspond well with their putative roles in spadefoot tadpole plasticity, direct functional assessment is needed of how polymorphisms in these genes influences spadefoot tadpoles’ ability to assess and respond to their environment (such direct functional assessment could involve, e.g., expression data or knockout studies). Second, because we sampled individuals from only two ponds (which may have been founded from the same source population; see section 2.2), additional studies are needed to determine whether these same candidate genes influence plasticity in other populations. Third, it is possible that some (or even all) of our candidate plasticity genes are not associated with plasticity *per se* but are instead associated with high fitness (perhaps only high‐fitness individuals are able to make an adaptive morph choice). Future research on more diverse population in more diverse ecological settings is needed to determine whether any of the candidate genes are involved in mediating plasticity *per se*.

Understanding the molecular mechanisms of plasticity and how these mechanisms evolve––and how their evolution, in turn, impacts plasticity's evolution––is an important frontier in ecology and evolution (Gilbert & Epel, 2015; Levis & Pfennig, 2020). While a growing number of studies have begun to illuminate how plasticity is regulated (Beldade et al., 2011; Lafuente & Beldade, 2019; Parsons et al., 2016; Projecto‐Garcia, Biddle, & Ragsdale, 2017; Richard, Le Trionnaire, Danchin, & Sentis, 2019; Serobyan & Sommer, 2017), much work remains. For instance, as noted in the Introduction, the prevailing view is that plasticity and its resulting phenotypes are regulated by different loci (Lafuente et al., 2018; Ørsted et al., 2018; Scheiner & Lyman, 1991; Schlichting & Pigliucci, 1993; Windig et al., 2004). However, there are few tests of this prediction. Our study provides such a test by demonstrating that different loci do indeed appear to be associated with morph versus adaptive plasticity (i.e., adaptive morph choice).

Moreover, most prior research aimed at identifying so‐called “switch” genes has focused on those genes at the start of a developmental sequence leading to production of an alternative phenotype (e.g., Corl et al., 2018; Miller, Longley, Hutchins, & Bauersachs, 2020; Ragsdale et al., 2013; Scoville & Pfrender, 2010; Suzuki & Nijhout, 2008). That is, most studies have focused on response and phenotype loci (the second stage of adaptive plasticity; see section 1). By contrast, few investigations into the genetic mechanisms underlying plasticity have examined how organisms assess their environment (the first stage of adaptive plasticity; see Introduction). Some of our candidate plasticity genes may be involved in this critically under‐studied first stage of plasticity (Table 2). In particular, genes associated with learning, behavior, and neuron function (GPR83, CLIP1, AFG3L2) might be crucial in assessment. This is perhaps not surprising given that the ability to assess the environment is needed not just for adaptive plasticity but for a whole range of day‐to‐day activities (Stevens, 2013). Future studies are needed to unravel how different genes and loci contribute to each of stage of adaptive plasticity.

In conclusion, our study utilized population genetic tools and natural ecological and phenotypic variation to identify loci contributing to plasticity in spadefoot toad tadpoles. Importantly, we were able to disentangle possible loci associated with the phenotypes produced by plasticity from loci associated plasticity *per se*. Our results thereby form the basis for future studies into the molecular mechanisms that mediate plasticity in spadefoots. More generally, these results illustrate how diverse loci might be deployed to mediate adaptive plasticity.

## CONFLICT OF INTEREST

The authors declare no conflict of interest.

## AUTHOR CONTRIBUTIONS

**Nicholas Levis:** Conceptualization (lead); Data curation (equal); Formal analysis (supporting); Funding acquisition (supporting); Investigation (equal); Methodology (equal); Project administration (lead); Resources (equal); Supervision (equal); Visualization (equal); Writing‐original draft (lead); Writing‐review & editing (lead). **Emily M.X. Reed:** Data curation (equal); Formal analysis (equal); Investigation (equal); Methodology (equal); Writing‐review & editing (supporting). **David Pfennig:** Conceptualization (equal); Funding acquisition (equal); Project administration (supporting); Resources (equal); Supervision (equal); Writing‐original draft (equal); Writing‐review & editing (equal). **Martha Overton Burford Reiskind:** Data curation (lead); Formal analysis (equal); Methodology (equal); Resources (equal); Writing‐original draft (supporting); Writing‐review & editing (supporting).

## ETHICAL APPROVAL

All procedures complied with all relevant ethical regulations, and our study protocol was approved by the University of North Carolina Institutional Animal Care and Use Committee (IACUC IDs 14‐297.0 and 17‐055.0). Field collections were conducted under scientific collection permit SP745794 provided by the Arizona Game and Fish Department.

## Supporting information

Table S1

## Data Availability

Data from this manuscript are available through Nicholas A. Levis’ Dryad account at the link https://datadryad.org/stash/share/1k5RwrMTUtVuSU9hNsOY0vgl6IA9IboiBGu2OVvZpok. These available data include post‐STACK analysis input data for PGDSpider and the list of aligned sequences containing outlier loci. Raw sequence data will be provided on request, as the data file size exceeds the limits of Dryad.
