# Supplementary material for: Identification of candidate loci for adaptive phenotypic plasticity in natural populations of spadefoot toads
Source: Ecol Evol. 2020 Jul 24;10(16):8976–88. doi: 10.1002/ece3.6602 (PMC7452772; doi:10.1002/ece3.6602)
Supplement: Supplementary file 1 — Table S1 [file ECE3-10-8976-s001.docx]

| Locus | Allele | Chorse | OHorse | CPO2-N | OPO2-N | Cboth | OBoth | Adaptive plasticity | Non-adaptive plasticity | Phorse | PPO2-N | Pmorph | Pplasticity | Qhorse | QPO2-N | Qmorph | Qplasticity | Mapped in association with trait |
| --- | --- | --- | --- | --- | --- | --- | --- | --- | --- | --- | --- | --- | --- | --- | --- | --- | --- | --- |
| 103612_94 | G | 82 | 84 | 40 | 34 | 122 | 118 | 116 | 124 | 0.4970 | 0.2211 | 1.0000 | 0.0570 | 0.5975 | 0.5717 | 1.0000 | 0.1056 | None |
| 103612_94 | C | 2 | 0 | 0 | 2 | 2 | 2 | 4 | 0 | --- | --- | --- | --- |  |  |  |  |  |
| 109213_50 | C | 86 | 84 | 42 | 37 | 128 | 121 | 123 | 126 | 0.0599 | 0.1116 | 0.7236 | 0.0071 | 0.1693 | 0.5717 | 0.8016 | 0.0360 | Plasticity |
| 109213_50 | T | 5 | 0 | 0 | 3 | 5 | 3 | 8 | 0 | --- | --- | --- | --- |  |  |  |  |  |
| 111746_82 | A | 85 | 84 | 40 | 34 | 125 | 118 | 119 | 124 | 0.1213 | 0.2211 | 0.6849 | 0.0294 | 0.2335 | 0.5717 | 0.8016 | 0.0577 | None |
| 111746_82 | T | 4 | 0 | 0 | 2 | 4 | 2 | 6 | 0 | --- | --- | --- | --- |  |  |  |  |  |
| 115957_93 | T | 85 | 82 | 42 | 40 | 127 | 122 | 125 | 124 | 0.6828 | 1.0000 | 0.6845 | 0.6839 | 0.7087 | 1.0000 | 0.8016 | 0.7730 | None |
| 115957_93 | C | 4 | 2 | 0 | 0 | 4 | 2 | 4 | 2 | --- | --- | --- | --- |  |  |  |  |  |
| 11813_26 | G | 83 | 84 | 42 | 35 | 125 | 119 | 118 | 126 | 0.1208 | 0.1027 | 1.0000 | 0.0070 | 0.2335 | 0.5717 | 1.0000 | 0.0360 | Plasticity |
| 11813_26 | A | 4 | 0 | 0 | 3 | 4 | 3 | 7 | 0 | --- | --- | --- | --- |  |  |  |  |  |
| 122954_12 | T | 89 | 80 | 39 | 36 | 128 | 116 | 125 | 119 | 0.0535 | 0.2448 | 0.7125 | 0.0143 | 0.1693 | 0.5717 | 0.8016 | 0.0360 | Plasticity |
| 122954_12 | C | 0 | 4 | 3 | 0 | 3 | 4 | 0 | 7 | --- | --- | --- | --- |  |  |  |  |  |
| 125043_49 | T | 89 | 82 | 35 | 36 | 124 | 118 | 125 | 117 | 0.2343 | 0.4321 | 1.0000 | 0.0998 | 0.3266 | 0.8276 | 1.0000 | 0.1652 | None |
| 125043_49 | A | 0 | 2 | 5 | 2 | 5 | 4 | 2 | 7 | --- | --- | --- | --- |  |  |  |  |  |
| 125054_73 | T | 85 | 86 | 42 | 36 | 127 | 122 | 121 | 128 | 0.1209 | 0.2225 | 0.6845 | 0.0144 | 0.2335 | 0.5717 | 0.8016 | 0.0360 | Plasticity |
| 125054_73 | G | 4 | 0 | 0 | 2 | 4 | 2 | 6 | 0 | --- | --- | --- | --- |  |  |  |  |  |
| 129589_86 | A | 80 | 82 | 42 | 36 | 122 | 118 | 116 | 124 | 0.1686 | 1.0000 | 0.1743 | 0.0997 | 0.2887 | 1.0000 | 0.5934 | 0.1652 | None |
| 129589_86 | C | 7 | 2 | 0 | 0 | 7 | 2 | 7 | 2 | --- | --- | --- | --- |  |  |  |  |  |
| 13128_19 | A | 76 | 84 | 41 | 33 | 117 | 117 | 109 | 125 | 0.0007 | 0.3300 | 0.0316 | 0.0003 | 0.0226 | 0.6975 | 0.3244 | 0.0060 | Morph, Plasticity |
| 13128_19 | G | 11 | 0 | 1 | 3 | 12 | 3 | 14 | 1 | --- | --- | --- | --- |  |  |  |  |  |
| 137655_81 | T | 89 | 84 | 38 | 34 | 127 | 118 | 123 | 122 | 0.2401 | 1.0000 | 0.4360 | 0.6837 | 0.3266 | 1.0000 | 0.8016 | 0.7730 | None |
| 137655_81 | G | 0 | 2 | 2 | 2 | 2 | 4 | 2 | 4 | --- | --- | --- | --- |  |  |  |  |  |
| 140796_70 | C | 82 | 84 | 40 | 36 | 122 | 120 | 118 | 124 | 0.0140 | 1.0000 | 0.0615 | 0.0597 | 0.0750 | 1.0000 | 0.3904 | 0.1072 | None |
| 140796_70 | T | 7 | 0 | 2 | 2 | 9 | 2 | 9 | 2 | --- | --- | --- | --- |  |  |  |  |  |
| 141320_81 | G | 85 | 86 | 40 | 34 | 125 | 120 | 119 | 126 | 0.1209 | 0.2211 | 0.6845 | 0.0143 | 0.2335 | 0.5717 | 0.8016 | 0.0360 | Plasticity |
| 141320_81 | T | 4 | 0 | 0 | 2 | 4 | 2 | 6 | 0 | --- | --- | --- | --- |  |  |  |  |  |
| 141320_82 | A | 85 | 86 | 40 | 34 | 125 | 120 | 119 | 126 | 0.1209 | 0.2211 | 0.6845 | 0.0143 | 0.2335 | 0.5717 | 0.8016 | 0.0360 | Plasticity |
| 141320_82 | G | 4 | 0 | 0 | 2 | 4 | 2 | 6 | 0 | --- | --- | --- | --- |  |  |  |  |  |
| 143748_44 | A | 89 | 82 | 38 | 38 | 127 | 120 | 127 | 120 | 0.2343 | 0.1174 | 0.6850 | 0.0144 | 0.3266 | 0.5717 | 0.8016 | 0.0360 | Plasticity |
| 143748_44 | T | 0 | 2 | 4 | 0 | 4 | 2 | 0 | 6 | --- | --- | --- | --- |  |  |  |  |  |
| 144818_85 | G | 83 | 84 | 38 | 36 | 121 | 120 | 119 | 122 | 0.1208 | 0.4933 | 0.6838 | 0.0294 | 0.2335 | 0.8766 | 0.8016 | 0.0577 | None |
| 144818_85 | T | 4 | 0 | 0 | 2 | 4 | 2 | 6 | 0 | --- | --- | --- | --- |  |  |  |  |  |
| 14665_28 | T | 83 | 84 | 38 | 34 | 121 | 118 | 117 | 122 | 0.1208 | 0.2332 | 0.6840 | 0.0294 | 0.2335 | 0.5717 | 0.8016 | 0.0577 | None |
| 14665_28 | G | 4 | 0 | 0 | 2 | 4 | 2 | 6 | 0 | --- | --- | --- | --- |  |  |  |  |  |
| 149541_73 | G | 81 | 82 | 42 | 36 | 123 | 118 | 117 | 124 | 0.1006 | 1.0000 | 0.1057 | 0.0597 | 0.2335 | 1.0000 | 0.4756 | 0.1072 | None |
| 149541_73 | T | 8 | 2 | 0 | 0 | 8 | 2 | 8 | 2 | --- | --- | --- | --- |  |  |  |  |  |
| 150827_60 | G | 89 | 83 | 31 | 36 | 120 | 119 | 125 | 114 | 0.4855 | 0.6618 | 1.0000 | 0.4323 | 0.5971 | 1.0000 | 1.0000 | 0.6344 | None |
| 150827_60 | A | 0 | 1 | 3 | 2 | 3 | 3 | 2 | 4 | --- | --- | --- | --- |  |  |  |  |  |
| 155974_32 | G | 83 | 84 | 38 | 33 | 121 | 117 | 116 | 122 | 0.1208 | 0.1101 | 1.0000 | 0.0143 | 0.2335 | 0.5717 | 1.0000 | 0.0360 | Plasticity |
| 155974_32 | C | 4 | 0 | 0 | 3 | 4 | 3 | 7 | 0 | --- | --- | --- | --- |  |  |  |  |  |
| 157502_81 | C | 81 | 80 | 42 | 40 | 123 | 120 | 121 | 122 | 0.7464 | 1.0000 | 0.5397 | 0.7491 | 0.7668 | 1.0000 | 0.8016 | 0.8218 | None |
| 157502_81 | A | 4 | 6 | 0 | 0 | 4 | 6 | 4 | 6 | --- | --- | --- | --- |  |  |  |  |  |
| 16123_30 | G | 83 | 86 | 40 | 34 | 123 | 120 | 117 | 126 | 0.1207 | 0.2211 | 0.6841 | 0.0136 | 0.2335 | 0.5717 | 0.8016 | 0.0360 | Plasticity |
| 16123_30 | C | 4 | 0 | 0 | 2 | 4 | 2 | 6 | 0 | --- | --- | --- | --- |  |  |  |  |  |
| 162331_44 | T | 85 | 82 | 42 | 40 | 127 | 122 | 125 | 124 | 0.6828 | 1.0000 | 0.6845 | 0.6839 | 0.7087 | 1.0000 | 0.8016 | 0.7730 | None |
| 162331_44 | C | 4 | 2 | 0 | 0 | 4 | 2 | 4 | 2 | --- | --- | --- | --- |  |  |  |  |  |
| 163218_83 | C | 85 | 82 | 42 | 40 | 127 | 122 | 125 | 124 | 0.6828 | 1.0000 | 0.6845 | 0.6839 | 0.7087 | 1.0000 | 0.8016 | 0.7730 | None |
| 163218_83 | A | 4 | 2 | 0 | 0 | 4 | 2 | 4 | 2 | --- | --- | --- | --- |  |  |  |  |  |
| 164909_83 | A | 87 | 80 | 39 | 38 | 126 | 118 | 125 | 119 | 0.0561 | 0.2424 | 0.7160 | 0.0143 | 0.1693 | 0.5717 | 0.8016 | 0.0360 | Plasticity |
| 164909_83 | G | 0 | 4 | 3 | 0 | 3 | 4 | 0 | 7 | --- | --- | --- | --- |  |  |  |  |  |
| 166790_81 | A | 85 | 82 | 36 | 34 | 121 | 116 | 119 | 118 | 0.2456 | 1.0000 | 0.4425 | 0.6836 | 0.3266 | 1.0000 | 0.8016 | 0.7730 | None |
| 166790_81 | T | 0 | 2 | 2 | 2 | 2 | 4 | 2 | 4 | --- | --- | --- | --- |  |  |  |  |  |
| 166790_82 | G | 85 | 82 | 36 | 34 | 121 | 116 | 119 | 118 | 0.2456 | 1.0000 | 0.4425 | 0.6836 | 0.3266 | 1.0000 | 0.8016 | 0.7730 | None |
| 166790_82 | A | 0 | 2 | 2 | 2 | 2 | 4 | 2 | 4 | --- | --- | --- | --- |  |  |  |  |  |
| 166790_87 | A | 85 | 80 | 36 | 34 | 121 | 114 | 119 | 116 | 0.2396 | 1.0000 | 0.4388 | 0.4462 | 0.3266 | 1.0000 | 0.8016 | 0.6386 | None |
| 166790_87 | C | 0 | 2 | 2 | 2 | 2 | 4 | 2 | 4 | --- | --- | --- | --- |  |  |  |  |  |
| 167858_59 | T | 78 | 78 | 37 | 37 | 115 | 115 | 115 | 115 | 0.6291 | 0.6156 | 0.3845 | 1.0000 | 0.7087 | 1.0000 | 0.8016 | 1.0000 | None |
| 167858_59 | C | 11 | 8 | 3 | 1 | 14 | 9 | 12 | 11 | --- | --- | --- | --- |  |  |  |  |  |
| 170340_25 | T | 87 | 73 | 41 | 34 | 128 | 107 | 121 | 114 | 0.0274 | 0.5926 | 0.0247 | 0.1074 | 0.1190 | 1.0000 | 0.2856 | 0.1709 | Morph |
| 170340_25 | C | 2 | 9 | 1 | 2 | 3 | 11 | 4 | 10 | --- | --- | --- | --- |  |  |  |  |  |
| 174208_15 | A | 78 | 78 | 34 | 38 | 112 | 116 | 116 | 112 | 0.2505 | 0.2332 | 0.1075 | 0.5979 | 0.3292 | 0.5717 | 0.4756 | 0.7730 | None |
| 174208_15 | G | 9 | 4 | 2 | 0 | 11 | 4 | 9 | 6 | --- | --- | --- | --- |  |  |  |  |  |
| 174502_84 | T | 75 | 80 | 40 | 40 | 115 | 120 | 115 | 120 | 0.2124 | 1.0000 | 0.2207 | 0.2207 | 0.3266 | 1.0000 | 0.6394 | 0.3416 | None |
| 174502_84 | G | 12 | 6 | 0 | 0 | 12 | 6 | 12 | 6 | --- | --- | --- | --- |  |  |  |  |  |
| 176019_68 | G | 91 | 80 | 40 | 38 | 131 | 118 | 129 | 120 | 0.0511 | 0.4949 | 0.4301 | 0.0137 | 0.1693 | 0.8766 | 0.8016 | 0.0360 | Plasticity |
| 176019_68 | A | 0 | 4 | 2 | 0 | 2 | 4 | 0 | 6 | --- | --- | --- | --- |  |  |  |  |  |
| 176019_70 | A | 91 | 80 | 40 | 38 | 131 | 118 | 129 | 120 | 0.0511 | 0.4949 | 0.4301 | 0.0137 | 0.1693 | 0.8766 | 0.8016 | 0.0360 | Plasticity |
| 176019_70 | G | 0 | 4 | 2 | 0 | 2 | 4 | 0 | 6 | --- | --- | --- | --- |  |  |  |  |  |
| 176103_71 | G | 85 | 82 | 40 | 38 | 125 | 120 | 123 | 122 | 0.6828 | 1.0000 | 0.6845 | 0.6838 | 0.7087 | 1.0000 | 0.8016 | 0.7730 | None |
| 176103_71 | A | 4 | 2 | 0 | 0 | 4 | 2 | 4 | 2 | --- | --- | --- | --- |  |  |  |  |  |
| 20520_85 | A | 85 | 84 | 42 | 36 | 127 | 120 | 121 | 126 | 0.1213 | 0.2225 | 0.6850 | 0.0294 | 0.2335 | 0.5717 | 0.8016 | 0.0577 | None |
| 20520_85 | C | 4 | 0 | 0 | 2 | 4 | 2 | 6 | 0 | --- | --- | --- | --- |  |  |  |  |  |
| 27227_74 | A | 87 | 84 | 40 | 36 | 127 | 120 | 123 | 124 | 0.1219 | 0.2341 | 0.6850 | 0.0296 | 0.2335 | 0.5717 | 0.8016 | 0.0577 | None |
| 27227_74 | G | 4 | 0 | 0 | 2 | 4 | 2 | 6 | 0 | --- | --- | --- | --- |  |  |  |  |  |
| 27680_85 | A | 89 | 84 | 38 | 34 | 127 | 118 | 123 | 122 | 0.4885 | 1.0000 | 0.6754 | 1.0000 | 0.5971 | 1.0000 | 0.8016 | 1.0000 | None |
| 27680_85 | C | 0 | 1 | 2 | 2 | 2 | 3 | 2 | 3 | --- | --- | --- | --- |  |  |  |  |  |
| 28134_59 | T | 82 | 85 | 41 | 37 | 123 | 122 | 119 | 126 | 0.0644 | 0.3535 | 0.3773 | 0.0343 | 0.1775 | 0.6975 | 0.8016 | 0.0645 | None |
| 28134_59 | C | 7 | 1 | 1 | 3 | 8 | 4 | 10 | 2 | --- | --- | --- | --- |  |  |  |  |  |
| 30117_86 | A | 81 | 86 | 42 | 36 | 123 | 122 | 117 | 128 | 0.0067 | 0.2225 | 0.1038 | 0.0008 | 0.0443 | 0.5717 | 0.4756 | 0.0110 | Plasticity |
| 30117_86 | T | 8 | 0 | 0 | 2 | 8 | 2 | 10 | 0 | --- | --- | --- | --- |  |  |  |  |  |
| 329681_70 | A | 85 | 80 | 42 | 34 | 127 | 114 | 119 | 122 | 0.0588 | 0.2098 | 0.0123 | 0.6840 | 0.1693 | 0.5717 | 0.2856 | 0.7730 | Morph |
| 329681_70 | G | 0 | 4 | 0 | 2 | 0 | 6 | 2 | 4 | --- | --- | --- | --- |  |  |  |  |  |
| 36393_80 | A | 82 | 64 | 30 | 35 | 112 | 99 | 117 | 94 | 0.0025 | 0.0225 | 0.3088 | 0.0001 | 0.0292 | 0.3182 | 0.7677 | 0.0049 | Plasticity |
| 36393_80 | G | 6 | 20 | 12 | 3 | 18 | 23 | 9 | 32 | --- | --- | --- | --- |  |  |  |  |  |
| 39320_63 | T | 84 | 71 | 34 | 37 | 118 | 108 | 121 | 105 | 0.0081 | 0.3580 | 0.1136 | 0.0027 | 0.0511 | 0.6975 | 0.4756 | 0.0232 | Plasticity |
| 39320_63 | G | 3 | 13 | 4 | 1 | 7 | 14 | 4 | 17 | --- | --- | --- | --- |  |  |  |  |  |
| 45428_84 | A | 87 | 86 | 42 | 36 | 129 | 122 | 123 | 128 | 0.1213 | 0.2225 | 0.6850 | 0.0294 | 0.2335 | 0.5717 | 0.8016 | 0.0577 | None |
| 45428_84 | C | 4 | 0 | 0 | 2 | 4 | 2 | 6 | 0 | --- | --- | --- | --- |  |  |  |  |  |
| 4743_89 | C | 58 | 70 | 32 | 26 | 90 | 96 | 84 | 102 | 0.0017 | 0.0704 | 0.1785 | 0.0003 | 0.0274 | 0.5492 | 0.5934 | 0.0060 | Plasticity |
| 4743_89 | T | 29 | 10 | 6 | 14 | 35 | 24 | 43 | 16 | --- | --- | --- | --- |  |  |  |  |  |
| 480108_86 | A | 33 | 50 | 26 | 28 | 59 | 78 | 61 | 76 | 0.0059 | 0.3405 | 0.0052 | 0.0764 | 0.0440 | 0.6975 | 0.2856 | 0.1328 | Morph |
| 480108_86 | G | 54 | 34 | 16 | 10 | 70 | 44 | 64 | 50 | --- | --- | --- | --- |  |  |  |  |  |
| 49345_6 | T | 86 | 78 | 41 | 36 | 127 | 114 | 122 | 119 | 0.0181 | 1.0000 | 0.0544 | 0.0193 | 0.0887 | 1.0000 | 0.3904 | 0.0445 | Plasticity |
| 49345_6 | C | 1 | 8 | 1 | 0 | 2 | 8 | 1 | 9 | --- | --- | --- | --- |  |  |  |  |  |
| 50679_85 | T | 87 | 84 | 36 | 36 | 123 | 120 | 123 | 120 | 0.2457 | 1.0000 | 0.4465 | 0.4465 | 0.3266 | 1.0000 | 0.8016 | 0.6386 | None |
| 50679_85 | G | 0 | 2 | 2 | 2 | 2 | 4 | 2 | 4 | --- | --- | --- | --- |  |  |  |  |  |
| 52193_80 | A | 85 | 80 | 42 | 38 | 127 | 118 | 123 | 122 | 0.6835 | 1.0000 | 0.6856 | 0.6838 | 0.7087 | 1.0000 | 0.8016 | 0.7730 | None |
| 52193_80 | G | 4 | 2 | 0 | 0 | 4 | 2 | 4 | 2 | --- | --- | --- | --- |  |  |  |  |  |
| 525106_74 | G | 87 | 82 | 38 | 34 | 125 | 116 | 121 | 120 | 0.2398 | 1.0000 | 0.4357 | 0.6837 | 0.3266 | 1.0000 | 0.8016 | 0.7730 | None |
| 525106_74 | A | 0 | 2 | 2 | 2 | 2 | 4 | 2 | 4 | --- | --- | --- | --- |  |  |  |  |  |
| 57316_85 | G | 87 | 82 | 40 | 38 | 127 | 120 | 125 | 122 | 0.6836 | 1.0000 | 0.6850 | 0.6841 | 0.7087 | 1.0000 | 0.8016 | 0.7730 | None |
| 57316_85 | A | 4 | 2 | 0 | 0 | 4 | 2 | 4 | 2 | --- | --- | --- | --- |  |  |  |  |  |
| 602213_75 | G | 89 | 76 | 40 | 36 | 129 | 112 | 125 | 116 | 0.0026 | 0.4965 | 0.0516 | 0.0016 | 0.0292 | 0.8766 | 0.3904 | 0.0184 | Plasticity |
| 602213_75 | T | 0 | 8 | 2 | 0 | 2 | 8 | 0 | 10 | --- | --- | --- | --- |  |  |  |  |  |
| 61852_75 | A | 87 | 82 | 34 | 36 | 121 | 118 | 123 | 116 | 0.2398 | 0.1153 | 0.6840 | 0.0143 | 0.3266 | 0.5717 | 0.8016 | 0.0360 | Plasticity |
| 61852_75 | C | 0 | 2 | 4 | 0 | 4 | 2 | 0 | 6 | --- | --- | --- | --- |  |  |  |  |  |
| 628171_94 | C | 79 | 78 | 38 | 34 | 117 | 112 | 113 | 116 | 0.0351 | 0.2633 | 0.4914 | 0.0097 | 0.1331 | 0.5722 | 0.8016 | 0.0360 | Plasticity |
| 628171_94 | T | 10 | 2 | 2 | 6 | 12 | 8 | 16 | 4 | --- | --- | --- | --- |  |  |  |  |  |
| 64949_86 | A | 66 | 44 | 24 | 30 | 90 | 74 | 96 | 68 | 0.0012 | 0.0681 | 0.0975 | 0.0002 | 0.0265 | 0.5492 | 0.4756 | 0.0052 | Plasticity |
| 64949_86 | G | 19 | 38 | 14 | 6 | 33 | 44 | 25 | 52 | --- | --- | --- | --- |  |  |  |  |  |
| 65737_67 | C | 82 | 84 | 42 | 37 | 124 | 121 | 119 | 126 | 0.0590 | 0.1116 | 0.7226 | 0.0070 | 0.1693 | 0.5717 | 0.8016 | 0.0360 | Plasticity |
| 65737_67 | T | 5 | 0 | 0 | 3 | 5 | 3 | 8 | 0 | --- | --- | --- | --- |  |  |  |  |  |
| 66007_69 | A | 87 | 84 | 40 | 32 | 127 | 116 | 119 | 124 | 0.2457 | 0.1414 | 0.0561 | 0.5365 | 0.3266 | 0.5717 | 0.3904 | 0.7485 | None |
| 66007_69 | G | 0 | 2 | 2 | 6 | 2 | 8 | 6 | 4 | --- | --- | --- | --- |  |  |  |  |  |
| 66007_70 | G | 87 | 84 | 40 | 32 | 127 | 116 | 119 | 124 | 0.2457 | 0.1414 | 0.0561 | 0.5365 | 0.3266 | 0.5717 | 0.3904 | 0.7485 | None |
| 66007_70 | A | 0 | 2 | 2 | 6 | 2 | 8 | 6 | 4 | --- | --- | --- | --- |  |  |  |  |  |
| 66072_36 | C | 46 | 14 | 6 | 22 | 52 | 36 | 68 | 20 | 0.0000 | 0.0001 | 0.1097 | 0.0000 | 0.0002 | 0.0168 | 0.4756 | 0.0000 | Plasticity |
| 66072_36 | T | 39 | 64 | 36 | 18 | 75 | 82 | 57 | 100 | --- | --- | --- | --- |  |  |  |  |  |
| 66229_35 | C | 89 | 85 | 37 | 37 | 126 | 122 | 126 | 122 | 0.4914 | 1.0000 | 0.7201 | 0.7201 | 0.5971 | 1.0000 | 0.8016 | 0.7977 | None |
| 66229_35 | T | 0 | 1 | 3 | 3 | 3 | 4 | 3 | 4 | --- | --- | --- | --- |  |  |  |  |  |
| 66421_32 | C | 81 | 82 | 36 | 38 | 117 | 120 | 119 | 118 | 0.6819 | 1.0000 | 0.4463 | 0.6837 | 0.7087 | 1.0000 | 0.8016 | 0.7730 | None |
| 66421_32 | T | 4 | 2 | 0 | 0 | 4 | 2 | 4 | 2 | --- | --- | --- | --- |  |  |  |  |  |
| 69337_35 | C | 74 | 70 | 40 | 34 | 114 | 104 | 108 | 110 | 0.5170 | 1.0000 | 0.5580 | 0.4307 | 0.6150 | 1.0000 | 0.8016 | 0.6344 | None |
| 69337_35 | A | 15 | 10 | 2 | 2 | 17 | 12 | 17 | 12 | --- | --- | --- | --- |  |  |  |  |  |
| 6994_11 | T | 85 | 86 | 20 | 36 | 105 | 122 | 121 | 106 | 0.1209 | 0.5402 | 0.4223 | 0.0330 | 0.2335 | 0.9392 | 0.8016 | 0.0632 | None |
| 6994_11 | C | 4 | 0 | 0 | 2 | 4 | 2 | 6 | 0 | --- | --- | --- | --- |  |  |  |  |  |
| 74500_85 | T | 85 | 86 | 42 | 36 | 127 | 122 | 121 | 128 | 0.1209 | 0.2225 | 0.6845 | 0.0144 | 0.2335 | 0.5717 | 0.8016 | 0.0360 | Plasticity |
| 74500_85 | A | 4 | 0 | 0 | 2 | 4 | 2 | 6 | 0 | --- | --- | --- | --- |  |  |  |  |  |
| 76279_44 | G | 81 | 85 | 38 | 35 | 119 | 120 | 116 | 123 | 0.1174 | 0.2400 | 0.7488 | 0.0191 | 0.2335 | 0.5717 | 0.8215 | 0.0445 | Plasticity |
| 76279_44 | A | 6 | 1 | 0 | 3 | 6 | 4 | 9 | 1 | --- | --- | --- | --- |  |  |  |  |  |
| 77782_49 | C | 77 | 84 | 38 | 33 | 115 | 117 | 110 | 122 | 0.0003 | 0.1543 | 0.1723 | 0.0001 | 0.0195 | 0.5717 | 0.5934 | 0.0049 | Plasticity |
| 77782_49 | G | 12 | 0 | 2 | 7 | 14 | 7 | 19 | 2 | --- | --- | --- | --- |  |  |  |  |  |
| 85910_51 | T | 87 | 76 | 41 | 36 | 128 | 112 | 123 | 117 | 0.1550 | 1.0000 | 0.3149 | 0.1023 | 0.2781 | 1.0000 | 0.7677 | 0.1652 | None |
| 85910_51 | C | 2 | 6 | 1 | 0 | 3 | 6 | 2 | 7 | --- | --- | --- | --- |  |  |  |  |  |
| 85910_70 | T | 87 | 76 | 41 | 36 | 128 | 112 | 123 | 117 | 0.1550 | 1.0000 | 0.3149 | 0.1023 | 0.2781 | 1.0000 | 0.7677 | 0.1652 | None |
| 85910_70 | G | 2 | 6 | 1 | 0 | 3 | 6 | 2 | 7 | --- | --- | --- | --- |  |  |  |  |  |
| 89487_25 | T | 80 | 84 | 40 | 36 | 120 | 120 | 116 | 124 | 0.0588 | 1.0000 | 0.1725 | 0.0997 | 0.1693 | 1.0000 | 0.5934 | 0.1652 | None |
| 89487_25 | C | 5 | 0 | 2 | 2 | 7 | 2 | 7 | 2 | --- | --- | --- | --- |  |  |  |  |  |
| 89816_20 | A | 66 | 73 | 36 | 36 | 102 | 109 | 102 | 109 | 0.1803 | 0.7383 | 0.1366 | 0.3246 | 0.2952 | 1.0000 | 0.5146 | 0.4890 | None |
| 89816_20 | T | 21 | 13 | 6 | 4 | 27 | 17 | 25 | 19 | --- | --- | --- | --- |  |  |  |  |  |
| 89816_21 | C | 66 | 73 | 36 | 36 | 102 | 109 | 102 | 109 | 0.1803 | 0.7383 | 0.1366 | 0.3246 | 0.2952 | 1.0000 | 0.5146 | 0.4890 | None |
| 89816_21 | T | 21 | 13 | 6 | 4 | 27 | 17 | 25 | 19 | --- | --- | --- | --- |  |  |  |  |  |
| 9089_86 | A | 85 | 80 | 42 | 38 | 127 | 118 | 123 | 122 | 0.6835 | 1.0000 | 0.6856 | 0.6838 | 0.7087 | 1.0000 | 0.8016 | 0.7730 | None |
| 9089_86 | T | 4 | 2 | 0 | 0 | 4 | 2 | 4 | 2 | --- | --- | --- | --- |  |  |  |  |  |
| 9287_42 | T | 82 | 78 | 41 | 37 | 123 | 115 | 119 | 119 | 0.5391 | 1.0000 | 0.5758 | 0.5711 | 0.6346 | 1.0000 | 0.8016 | 0.7730 | None |
| 9287_42 | C | 7 | 4 | 1 | 1 | 8 | 5 | 8 | 5 | --- | --- | --- | --- |  |  |  |  |  |
| 95380_86 | T | 83 | 82 | 40 | 40 | 123 | 122 | 123 | 122 | 0.6822 | 1.0000 | 0.6838 | 0.6838 | 0.7087 | 1.0000 | 0.8016 | 0.7730 | None |
| 95380_86 | G | 4 | 2 | 0 | 0 | 4 | 2 | 4 | 2 | --- | --- | --- | --- |  |  |  |  |  |
| 97986_79 | T | 85 | 86 | 42 | 36 | 127 | 122 | 121 | 128 | 0.1209 | 0.2225 | 0.6845 | 0.0144 | 0.2335 | 0.5717 | 0.8016 | 0.0360 | Plasticity |
| 97986_79 | G | 4 | 0 | 0 | 2 | 4 | 2 | 6 | 0 | --- | --- | --- | --- |  |  |  |  |  |
| 99834_86 | C | 85 | 82 | 42 | 40 | 127 | 122 | 125 | 124 | 0.6828 | 1.0000 | 0.6845 | 0.6839 | 0.7087 | 1.0000 | 0.8016 | 0.7730 | None |
| 99834_86 | G | 4 | 2 | 0 | 0 | 4 | 2 | 4 | 2 | --- | --- | --- | --- |  |  |  |  |  |
| 123382_47 | T | 63 | 53 | 33 | 18 | 96 | 71 | 81 | 86 | 0.1521 | 0.0018 | 0.0023 | 0.6920 | 0.2781 | 0.0397 | 0.2582 | 0.7742 | Morph |
| 123382_47 | C | 25 | 35 | 7 | 20 | 32 | 55 | 45 | 42 | --- | --- | --- | --- |  |  |  |  |  |
| 135603_36 | C | 59 | 51 | 21 | 17 | 80 | 68 | 76 | 72 | 0.3434 | 0.4916 | 0.1964 | 0.7962 | 0.4409 | 0.8766 | 0.6032 | 0.8472 | None |
| 135603_36 | T | 28 | 33 | 17 | 21 | 45 | 54 | 49 | 50 | --- | --- | --- | --- |  |  |  |  |  |
| 140565_58 | T | 51 | 36 | 21 | 25 | 72 | 61 | 76 | 57 | 0.0219 | 0.2381 | 0.2007 | 0.0103 | 0.1029 | 0.5717 | 0.6032 | 0.0360 | Plasticity |
| 140565_58 | G | 34 | 50 | 17 | 11 | 51 | 61 | 45 | 67 | --- | --- | --- | --- |  |  |  |  |  |
| 142120_80 | G | 65 | 40 | 18 | 20 | 83 | 60 | 85 | 58 | 0.0008 | 0.8187 | 0.0099 | 0.0019 | 0.0226 | 1.0000 | 0.2856 | 0.0199 | Morph, Plasticity |
| 142120_80 | T | 22 | 42 | 20 | 18 | 42 | 60 | 40 | 62 | --- | --- | --- | --- |  |  |  |  |  |
| 148855_19 | T | 48 | 31 | 16 | 25 | 64 | 56 | 73 | 47 | 0.0146 | 0.0729 | 0.3791 | 0.0016 | 0.0750 | 0.5492 | 0.8016 | 0.0184 | Plasticity |
| 148855_19 | C | 38 | 53 | 24 | 15 | 62 | 68 | 53 | 77 | --- | --- | --- | --- |  |  |  |  |  |
| 152023_16 | C | 43 | 38 | 24 | 14 | 67 | 52 | 57 | 62 | 0.4500 | 0.1072 | 0.1006 | 0.8006 | 0.5650 | 0.5717 | 0.4756 | 0.8472 | None |
| 152023_16 | T | 44 | 50 | 16 | 22 | 60 | 72 | 66 | 66 | --- | --- | --- | --- |  |  |  |  |  |
| 152023_43 | A | 44 | 38 | 24 | 14 | 68 | 52 | 58 | 62 | 0.4500 | 0.1072 | 0.0791 | 0.8021 | 0.5650 | 0.5717 | 0.4664 | 0.8472 | None |
| 152023_43 | G | 44 | 50 | 16 | 22 | 60 | 72 | 66 | 66 | --- | --- | --- | --- |  |  |  |  |  |
| 164226_89 | C | 40 | 56 | 28 | 26 | 68 | 82 | 66 | 84 | 0.0018 | 1.0000 | 0.0127 | 0.0061 | 0.0274 | 1.0000 | 0.2856 | 0.0360 | Morph, Plasticity |
| 164226_89 | G | 47 | 24 | 12 | 12 | 59 | 36 | 59 | 36 | --- | --- | --- | --- |  |  |  |  |  |
| 172194_52 | T | 67 | 66 | 21 | 18 | 88 | 84 | 85 | 87 | 0.6065 | 0.8210 | 0.8945 | 0.5948 | 0.7065 | 1.0000 | 0.9514 | 0.7730 | None |
| 172194_52 | C | 25 | 20 | 19 | 20 | 44 | 40 | 45 | 39 | --- | --- | --- | --- |  |  |  |  |  |
| 2047_97 | G | 49 | 68 | 24 | 20 | 73 | 88 | 69 | 92 | 0.0059 | 0.6364 | 0.0622 | 0.0078 | 0.0440 | 1.0000 | 0.3904 | 0.0360 | Plasticity |
| 2047_97 | C | 37 | 20 | 14 | 16 | 51 | 36 | 53 | 34 | --- | --- | --- | --- |  |  |  |  |  |
| 2047_98 | C | 50 | 68 | 24 | 20 | 74 | 88 | 70 | 92 | 0.0092 | 0.6364 | 0.0825 | 0.0113 | 0.0549 | 1.0000 | 0.4664 | 0.0360 | Plasticity |
| 2047_98 | G | 36 | 20 | 14 | 16 | 50 | 36 | 52 | 34 | --- | --- | --- | --- |  |  |  |  |  |
| 2387_15 | G | 46 | 51 | 30 | 17 | 76 | 68 | 63 | 81 | 0.2240 | 0.0012 | 0.5248 | 0.0050 | 0.3266 | 0.0353 | 0.8016 | 0.0335 | Plasticity |
| 2387_15 | A | 44 | 33 | 8 | 23 | 52 | 56 | 67 | 41 | --- | --- | --- | --- |  |  |  |  |  |
| 2387_53 | T | 45 | 51 | 30 | 17 | 75 | 68 | 62 | 81 | 0.1720 | 0.0012 | 0.6113 | 0.0034 | 0.2901 | 0.0353 | 0.8016 | 0.0274 | Plasticity |
| 2387_53 | G | 45 | 33 | 8 | 23 | 53 | 56 | 68 | 41 | --- | --- | --- | --- |  |  |  |  |  |
| 2387_75 | C | 46 | 51 | 30 | 17 | 76 | 68 | 63 | 81 | 0.2240 | 0.0012 | 0.5248 | 0.0050 | 0.3266 | 0.0353 | 0.8016 | 0.0335 | Plasticity |
| 2387_75 | G | 44 | 33 | 8 | 23 | 52 | 56 | 67 | 41 | --- | --- | --- | --- |  |  |  |  |  |
| 26065_81 | T | 53 | 64 | 26 | 22 | 79 | 86 | 75 | 90 | 0.0807 | 0.6423 | 0.2945 | 0.0673 | 0.2171 | 1.0000 | 0.7677 | 0.1188 | None |
| 26065_81 | G | 36 | 24 | 14 | 16 | 50 | 40 | 52 | 38 | --- | --- | --- | --- |  |  |  |  |  |
| 27065_38 | C | 65 | 65 | 19 | 20 | 84 | 85 | 85 | 84 | 1.0000 | 1.0000 | 0.8940 | 1.0000 | 1.0000 | 1.0000 | 0.9514 | 1.0000 | None |
| 27065_38 | T | 24 | 23 | 19 | 18 | 43 | 41 | 42 | 42 | --- | --- | --- | --- |  |  |  |  |  |
| 32604_52 | C | 57 | 40 | 16 | 21 | 73 | 61 | 78 | 56 | 0.0129 | 0.2567 | 0.2029 | 0.0049 | 0.0730 | 0.5717 | 0.6032 | 0.0335 | Plasticity |
| 32604_52 | T | 28 | 44 | 24 | 17 | 52 | 61 | 45 | 68 | --- | --- | --- | --- |  |  |  |  |  |
| 32604_54 | G | 60 | 40 | 15 | 21 | 75 | 61 | 81 | 55 | 0.0056 | 0.2506 | 0.1274 | 0.0022 | 0.0440 | 0.5717 | 0.5141 | 0.0211 | Plasticity |
| 32604_54 | A | 28 | 46 | 23 | 17 | 51 | 63 | 45 | 69 | --- | --- | --- | --- |  |  |  |  |  |
| 3754_77 | A | 76 | 56 | 20 | 20 | 96 | 76 | 96 | 76 | 0.0019 | 0.8251 | 0.0239 | 0.0112 | 0.0274 | 1.0000 | 0.2856 | 0.0360 | Morph, Plasticity |
| 3754_77 | G | 14 | 32 | 20 | 18 | 34 | 50 | 32 | 52 | --- | --- | --- | --- |  |  |  |  |  |
| 42984_21 | T | 71 | 63 | 24 | 35 | 95 | 98 | 106 | 87 | 0.2990 | 0.0100 | 0.5674 | 0.0147 | 0.3883 | 0.1620 | 0.8016 | 0.0360 | Plasticity |
| 42984_21 | G | 19 | 25 | 16 | 5 | 35 | 30 | 24 | 41 | --- | --- | --- | --- |  |  |  |  |  |
| 42984_22 | A | 71 | 63 | 24 | 35 | 95 | 98 | 106 | 87 | 0.2237 | 0.0100 | 0.6656 | 0.0095 | 0.3266 | 0.1620 | 0.8016 | 0.0360 | Plasticity |
| 42984_22 | G | 18 | 25 | 16 | 5 | 34 | 30 | 23 | 41 | --- | --- | --- | --- |  |  |  |  |  |
| 50174_30 | G | 59 | 39 | 27 | 24 | 86 | 63 | 83 | 66 | 0.0062 | 1.0000 | 0.0205 | 0.0289 | 0.0440 | 1.0000 | 0.2856 | 0.0577 | Morph |
| 50174_30 | A | 30 | 47 | 13 | 12 | 43 | 59 | 42 | 60 | --- | --- | --- | --- |  |  |  |  |  |
| 50174_65 | A | 59 | 39 | 27 | 24 | 86 | 63 | 83 | 66 | 0.0062 | 1.0000 | 0.0205 | 0.0289 | 0.0440 | 1.0000 | 0.2856 | 0.0577 | Morph |
| 50174_65 | T | 30 | 47 | 13 | 12 | 43 | 59 | 42 | 60 | --- | --- | --- | --- |  |  |  |  |  |
| 50815_43 | A | 61 | 41 | 16 | 25 | 77 | 66 | 86 | 57 | 0.0062 | 0.0649 | 0.2549 | 0.0006 | 0.0440 | 0.5492 | 0.7201 | 0.0098 | Plasticity |
| 50815_43 | G | 29 | 47 | 22 | 13 | 51 | 60 | 42 | 69 | --- | --- | --- | --- |  |  |  |  |  |
| 51475_28 | T | 63 | 52 | 26 | 32 | 89 | 84 | 95 | 78 | 0.1404 | 0.0475 | 0.7796 | 0.0238 | 0.2644 | 0.4881 | 0.8471 | 0.0537 | None |
| 51475_28 | C | 23 | 32 | 12 | 4 | 35 | 36 | 27 | 44 | --- | --- | --- | --- |  |  |  |  |  |
| 618607_5 | T | 61 | 42 | 20 | 26 | 81 | 68 | 87 | 62 | 0.0311 | 0.2405 | 0.3038 | 0.0100 | 0.1300 | 0.5717 | 0.7677 | 0.0360 | Plasticity |
| 618607_5 | C | 30 | 42 | 18 | 12 | 48 | 54 | 42 | 60 | --- | --- | --- | --- |  |  |  |  |  |
| 71562_82 | A | 43 | 46 | 24 | 22 | 67 | 68 | 65 | 70 | 0.6530 | 1.0000 | 0.7090 | 0.6166 | 0.7087 | 1.0000 | 0.8016 | 0.7730 | None |
| 71562_82 | G | 47 | 42 | 16 | 16 | 63 | 58 | 63 | 58 | --- | --- | --- | --- |  |  |  |  |  |
| 71562_84 | G | 45 | 46 | 24 | 22 | 69 | 68 | 67 | 70 | 0.7668 | 1.0000 | 0.9009 | 0.8022 | 0.7806 | 1.0000 | 0.9514 | 0.8472 | None |
| 71562_84 | A | 45 | 42 | 16 | 16 | 61 | 58 | 61 | 58 | --- | --- | --- | --- |  |  |  |  |  |
| 71562_86 | A | 46 | 46 | 24 | 22 | 70 | 68 | 68 | 70 | 0.8821 | 1.0000 | 1.0000 | 0.9003 | 0.8899 | 1.0000 | 1.0000 | 0.9333 | None |
| 71562_86 | C | 44 | 42 | 16 | 16 | 60 | 58 | 60 | 58 | --- | --- | --- | --- |  |  |  |  |  |
| 80924_26 | A | 42 | 55 | 26 | 20 | 68 | 75 | 62 | 81 | 0.0365 | 0.2580 | 0.3193 | 0.0126 | 0.1331 | 0.5717 | 0.7677 | 0.0360 | Plasticity |
| 80924_26 | G | 48 | 33 | 14 | 20 | 62 | 53 | 68 | 47 | --- | --- | --- | --- |  |  |  |  |  |
| 80924_27 | T | 42 | 55 | 25 | 20 | 67 | 75 | 62 | 80 | 0.0365 | 0.1766 | 0.3787 | 0.0121 | 0.1331 | 0.5717 | 0.8016 | 0.0360 | Plasticity |
| 80924_27 | A | 48 | 33 | 13 | 20 | 61 | 53 | 68 | 46 | --- | --- | --- | --- |  |  |  |  |  |
| 80924_33 | A | 40 | 53 | 25 | 20 | 65 | 73 | 60 | 78 | 0.0348 | 0.1766 | 0.3757 | 0.0116 | 0.1331 | 0.5717 | 0.8016 | 0.0360 | Plasticity |
| 80924_33 | T | 48 | 33 | 13 | 20 | 61 | 53 | 68 | 46 | --- | --- | --- | --- |  |  |  |  |  |
| 81883_88 | A | 55 | 44 | 34 | 26 | 87 | 70 | 79 | 78 | 0.1682 | 0.0467 | 0.0348 | 1.0000 | 0.2887 | 0.4881 | 0.3273 | 1.0000 | Morph |
| 81883_88 | C | 33 | 42 | 4 | 12 | 37 | 54 | 45 | 46 | --- | --- | --- | --- |  |  |  |  |  |
| 81883_89 | C | 55 | 44 | 34 | 26 | 89 | 70 | 81 | 78 | 0.1682 | 0.0467 | 0.0253 | 0.8955 | 0.2887 | 0.4881 | 0.2856 | 0.9333 | Morph |
| 81883_89 | A | 33 | 42 | 4 | 12 | 37 | 54 | 45 | 46 | --- | --- | --- | --- |  |  |  |  |  |
| 86133_50 | A | 63 | 45 | 21 | 27 | 84 | 72 | 90 | 66 | 0.0392 | 0.3525 | 0.2946 | 0.0185 | 0.1384 | 0.6975 | 0.7677 | 0.0444 | Plasticity |
| 86133_50 | G | 26 | 37 | 17 | 13 | 43 | 50 | 39 | 54 | --- | --- | --- | --- |  |  |  |  |  |
| 86133_74 | T | 63 | 45 | 21 | 27 | 84 | 72 | 90 | 66 | 0.0246 | 0.3525 | 0.1900 | 0.0121 | 0.1110 | 0.6975 | 0.6032 | 0.0360 | Plasticity |
| 86133_74 | C | 24 | 37 | 17 | 13 | 41 | 50 | 37 | 54 | --- | --- | --- | --- |  |  |  |  |  |
| 95875_86 | C | 69 | 60 | 32 | 34 | 101 | 94 | 103 | 92 | 0.2413 | 0.7695 | 0.4701 | 0.1931 | 0.3266 | 1.0000 | 0.8016 | 0.3031 | None |
| 95875_86 | A | 21 | 28 | 8 | 6 | 29 | 34 | 27 | 36 | --- | --- | --- | --- |  |  |  |  |  |
